# Physico-chemical characterization of food grade natural spring salt from the Central Anatolia region of Turkey and investigation of its microplastic content

**DOI:** 10.1007/s13197-024-05942-0

**Published:** 2024-02-26

**Authors:** Ogün Bozkaya, Yaşar Aluç

**Affiliations:** 1 Scientific and Technological Researches Application and Research Center, Science and Engineering Laboratories, Kırıkkale University, 71450 Kırıkkale, Turkey; 2 Organic Agriculture Programme, Department of Crop and Animal Production, Delice Vocational School, Kırıkkale University, 71450 Kırıkkale, Turkey

**Keywords:** Delice salt, Spring salt, Heavy metals, Trace elements, Microplastics, Food characterization

## Abstract

Delice salt is a natural spring salt obtained from the evaporation of spring water from the Central Anatolia region of Turkey and is a food grade salt with a geographical indication certificate. The aim of this study was to evaluate the compliance of Delice natural spring salt (Delice NSS), a commercial product, with food grade criteria by physicochemical characterizations and to investigate whether it contains microplastics. The NaCl%, moisture suspended solids and acid insoluble matter contents of Delice NSS were analyzed and determined as 98.79% (w/w), 0.301% (w/w), 0.16% (w/w) and 0.01% (w/w), respectively. Sodium, other minerals, and heavy metals were analyzed by inductively coupled plasma-optical emission (ICP-OES) spectrometry. In addition, fluoride, nitrate, and sulphate ions were analyzed by ion chromatography (IC). The chemical and thermal properties of Delice NSS were characterized by FTIR, X-ray fluorescence (XRF) and thermogravimetric analyzer (TGA). Moreover, Delice NSS was investigated whether it contains microplastics and some particles were detected under fluorescence microscopy. However, it was concluded that particles were not microplastics after TGA and FTIR analyses. The results revealed that Delice NSS does not carry a risk in terms of physicochemical properties as a foodstuff.

**Supplementary Information:**

The online version contains supplementary material available at 10.1007/s13197-024-05942-0.

## Introduction

Sodium chloride (NaCl), or most commonly table salt, is a very important mineral that has been known to exist since ancient times and has a vital importance for humanity. This precious mineral has gained a social, religious, and even political identity in various parts of the world since ancient times and has affected societies. Salt is a food ingredient used in almost all ready-to-eat foods due to its flavor enhancing, unpleasant taste masking and preservative properties. Salt, which is an indispensable part of the table, has had different uses from past to present. Salt, which was used only to increase the flavor of foods and to keep them intact for a long time, has now become a raw material used in various industries such as chemistry, medicine, cosmetics, petroleum, energy, and textiles. In addition, approximately 30% of the salt produced in the world is dumped on highways to prevent icing (Sedat 2012). In the world, salt is found both dissolved in water (such as sea, lake, spring) and in the form of rock salt. Although technology has been added at different rates according to the development level of the countries, the methods used in salt production today are the same or very similar to the methods used in ancient times. Rock salt, scientifically known as halite, has been extracted from underground salt mines by classical mining techniques. Today, very few rock salt sources can be used directly without any purification. However, if it is mixed with other substances, it is made suitable for use after first being melted and then recrystallized. Another of the main salt production methods used since ancient times has been the processing of salty sea, lake, and some spring waters. The main method used to obtain salt from salt waters is evaporation. Seas and lakes are of great importance in the production of salt from surface waters. However, such waters may contain many dangerous ions and pollutants for human health. For this reason, the salts obtained from these waters are presented to the consumer after going through various separation, purification and even adding stages. For example, after purification of food-grade salts from such sources, anti-caking agents such as potassium ferrocyanide, sodium ferrocyanide, sodium aluminum silicate are added (Ercoşkun 2022). It has been reported in the literature that the excess of ferrocyanide compounds taken with these consumed salts is harmful to health. On the other hand, recent research has revealed that sea, lake, and rock salts may contain heavy metals such as mercury (Hg), arsenic (As), cadmium (Cd), copper (Cu) and lead (Pb) (Ercoşkun 2022). Therefore, direct consumption by humans is not recommended due to the impurities of these salts. In addition to heavy metals and additives, very small pieces of waste plastic called microplastics (MPs) have been detected especially in sea and lake salts in recent years. MPs are particles of plastic waste produced from polymers such as polyethylene (PE), nylon, polystyrene (PS), polypropylene (PP), polyethylene terephthalate (PET) and polyvinyl chloride (PVC), which are widely used in land and maritime industries. Micro and nano-sized plastic particles, which are formed as a result of the breakdown of waste plastics due to weather conditions, mechanical wear, sun rays and microorganisms, are carried to nature, seas, lakes and streams much further away by the effect of the wind (Ali et al. 2023). Since there is no enzymatic way to break down MPs, which are synthetic polymers, in any living creature, both terrestrial and aquatic, the ingested microplastics are never digested or absorbed. Therefore, it is an undeniable fact that MPs threaten both underwater ecosystems and human health.

Unlike sea and lake salts, natural spring salts that come to the surface by themselves are mostly offered to consumers without the need for any separation and purification process, since it is claimed that they do not contain synthetic pollutants such as microplastics (O'Connor 2023). Natural spring salts are the salts formed as a result of the evaporation of the salty water that comes out of the earth spontaneously by dissolving and incorporating the rock salts as it passes through the salty rock fragments of the underground spring waters. Salt obtained from such sources is also called evaporated salt. Minerals in the structure of spring salts are directly related to the region where they are located and to the different minerals carried by the spring waters in that region. Natural spring salt deposits are found in a few countries around the world, so it is a valuable product. It has natural spring salt deposits along the Bolivian and Peruvian Andes Mountains. Turkey, which has an important salt reserve throughout the world, has natural spring salt reserves extending from the Central Anatolian Region to the Eastern Anatolia Region. Natural spring salt extracted in Delice district of Kırıkkale province located in Central Anatolia region of Turkey; It is a natural spring salt that is kept under the sun in the pools located 500 m from the Plural saltworks after the underground waters of the Delice district come to the surface and crystallized in the natural environment and without being refined. This salt, called Delice natural spring salt, was registered by Kırıkkale Chamber of Commerce and Industry in February 2022 and received a Geographical Indication Certificate (Registration No: 1024).

Due to the increasing interest of the world's leading restaurants in natural spring salt in recent years, the physical and chemical properties of these salts have been the subject of research. Therefore, this study primarily aimed to fill the gaps in the literature by physicochemical characterization of Delice natural spring salt. In addition, due to the exponential increase in microplastic monitoring data in recent years, microplastic pollution was evaluated in this study.

## Materials and methods

Delice natural spring salt (Delice NSS), declared to be additive-free, was obtained from a local market (Mayi Salt, Kırıkkale, Turkey). NaCl (≥ 99,5%) and all other chemicals (reagent grade) used in the analyzes were purchased from Merck (Darmstadt, Germany).

### Physico-chemical characterizations

#### NaCl content, total solids, suspended solids and acid insoluble matter analysis

The percentage of NaCl of Delice NSS was calculated according to ASTM D632-18. Moreover, total solid, suspended solid matter and acid insoluble matter analyses were performed and detailed test methods are given in the supplementary data file.

#### Inductively coupled plasma-optical emission spectroscopy analysis

Qualitative and quantitative analysis of heavy metals such as Arsenic (As), Mercury (Hg), Copper (Cu), Lead (Pb), Antimony (Sb) and other minerals including Na of the salt sample was performed by Inductively Coupled Plasma-Optical Emission Spectroscopy (ICP-OES, Spectroblue) device. Before the analysis, approximately 1.0 g of salt samples were placed in a mixture of 45 mL of concentrated HNO_3_ and 5 mL of dH_2_O and subjected to combustion in a microwave device (CEM MARS6) under certain conditions.

#### Ion chromatography analysis

Approximately 1.0 g of salt sample was dissolved in 100 mL dH_2_O (TIP-I, ultra-pure distilled water) by sonication for 30 min and then passed through a 0.45 µm injector filter. Bromate (BrO_3_^−^), nitrate (NO_3_^−^), nitrite (NO_2_^−^), chlorate (ClO_3_^−^), chlorite (ClO_2_^−^), phosphate (PO_4_^−3^), sulfate (SO_4_^2−^), fluoride (F^−^), and bromide (Br^−^) were performed with an ion chromatography device (IC, Prominence HIC-20A Super, Shimadzu). Shimpack ICSA2 (250 mm × 4.0 mm, 9 μm) column was used for anion analysis.

#### CHNS elemental analysis

Total carbon (TC), nitrogen (TN), hydrogen (TH) and sulfur (TS) analyzes of the salt samples were performed by CHNS elemental analyzer (Elementar Vario Microcub). The samples were placed in silver capsules and given to the device. Analyses were performed at combustion and reduction column temperatures of 1150 °C and 850 °C, respectively, and flow rates of high purity He and O_2_ gases of 200 mL/min and 14 mL/min, respectively.

#### Energy dispersive X-ray fluorescence analysis

Approximately 4 g of the salt samples were ground to fine powder in a mortar. The samples were then compressed into a 32 mm diameter disc and analyzed by an energy dispersive X-ray fluorescence (ED-XRF, Spectro Xepos, AMETEK) spectrometer (He gas flow rate 90 mL/min, voltage 39.8 kV and current 0.9 mA).

#### Fourier transform infrared spectroscopy analysis

Chemical bond characterization of the salt samples was performed by Fourier Transform Infrared Spectroscopy (FTIR, Breker Vertex 70 V, USA). The samples were analyzed in the mid-infrared (MIR, 400–4000 cm^−1^) region using the attenuated total reflection (ATR) attachment (resolution 4 cm^−1^, scan speed 32 cm^−1^).

#### Thermal analysis

Thermal characterization of NaCl and Delice NSS was carried out with a thermogravimetric analyzer (TGA, Q500, TA Instruments) in the temperature range of 30–1000 °C (heating rate: 20 °C /min) in nitrogen gas (N_2_, flow rate: 50 mL/min) atmosphere.

#### Microplastic analysis

Approximately 10 g of salt sample was completely dissolved in 50 mL of ultrapure water previously passed through a 0.2 μm pore size filter. The prepared solution was passed through 0.45 μm filter paper using a vacuum filtration apparatus placed in a flow cabinet. Immediately afterwards, the filter paper was examined with a fluorescence microscope (Leica, DM5000 B) at 50 × magnification.

## Results and discussion

### Determination of physical content

The NaCl content of Delice NSS salt was calculated as 98.79%. According to European Commission (EC), World Health Organization (WHO) and Turkish Food Codex (TFC), NaCl content cannot be less than 97% on dry matter basis, excluding additives. Therefore, in this respect, Delice NSS is included in the food grade salt category.

The amount of substance (moisture) and total solid mass evaporated from Delice NSS were determined as 0.301% (w/w) and 99.699% (w/w), respectively. Hassan et al. reported that the moisture content of Himalayan salts collected from different mines ranged from 0.03 to 0.09% (ul Hassan et al. 2017). In addition, the researchers stated that the moisture content of the same salts in previous studies was in the range of 0.55%-0.65% and the unrefined form was in the range of 0.9–1.2%. According to the TFC, the amount of moisture in underground spring and sea salts must be at most 2% by mass, and 0.5% in other salts. In addition, in the salt specifications report published by the World Food Program (WFP), the maximum moisture content is specified as 3.0%.

The amounts of water-insoluble (suspended solid matter) in the Delice NSS were determined as 0.16% (w/w). According to TFC, the amount of water-insoluble matter in processed salts is 0.5% by mass, while this value is specified as 0.2% in the WFP report. Hassan et al. reported that the amount of water-insoluble matter in Himalayan salt samples was between 0.02 and 2.8% and these values were within the range determined by the Codex Alimentarius Commission. (ul Hassan et al. 2017). Santhanakrishnan et al. (Santhanakrishnan et al. 2015). reported an average of 0.74% water-insoluble matter in solar evaporated salt samples in Tamilnadu, India.

The amounts of acid-insoluble substances in the Delice NSS were determined as % 0.01 (w/w). According to TFC and WFP, the amount of acid-insoluble matter is limited to a maximum of 0.5% and 0.15%, respectively.

### Sodium and other trace element analysis by ICP-OES

The sodium content of Delice NSS was determined as 355 g/L. Sodium is an essential mineral for the human body and is required for normal cell function and neurotransmission. However, some studies have reported that excessive sodium combined with excessive salt intake has negative effects on health, such as high blood pressure, cardiovascular diseases, stomach cancer and impaired kidney function (Malta et al. 2018). For this reason, research institutions and food industries continue to develop different strategies to reduce the sodium level of foods by working together. In recent years, interest in sea, rock and spring salts has increased considerably due to their low sodium content and natural minerals found in their structures. In addition, the Na content by mass of Delice NSS was calculated as 34.44 ± 0.98% (w/w). This value is lower than the Na content of refined table salt (39.12%). The Na content of the world-famous Himalayan (Himalayan Mountains), Maldon (England) and LG Celtic (France) salts are 36.81%, 38.30% and 33.30%, respectively. According to the results of ICP-OES, in addition to Na; Li, Mg, K, Ca, Sr, V and Zn minerals were determined in the Delice NSS, and the results are given in Table 1.

**Table 1:** ICP-OES results of some minerals and trace elements

Element	Concentration
Lithium (Li)	230.1 ± 26.2 µg/kg
Magnesium (Mg)	48.26 ± 7.16 mg/kg
Potassium (K)	11.22 ± 5.6 mg/kg
Calcium (Ca)	6160.16 ± 140.11 mg/kg
Strontium (Sr)	37.11 ± 2.9 mg/kg
Vanadium (V)	350.2 ± 41.6 µg/kg
Copper (Cu)	< 0.013 mg/kg
Zinc (Zn)	44.0 ± 3.2 mg/kg
Arsenic (As)	< 0.0142 mg/kg
Cadmium (Cd)	< 0.038 mg/kg
Mercury (Hg)	< 0.0019 mg/kg
Lead (Pb)	< 0.018 mg/kg

Lithium (Li), a natural trace element, is especially important for cognitive health. Numerous studies reports that lithium salts are effective in the treatment, long-term maintenance of mood and prophylaxis of manic-depressive psychosis (Nielsen 1998). Based on monogastric rat studies, the daily Li requirement for humans is between 200 and 600 µg (Gimeno et al. 2018). The Li concentration in Delice NSS is 230.1 ± 26.2 µg/kg. Ercoşkun (Ercoşkun 2022) reported that he detected Li only in Guérande flake sea salt, in his research on 18 different salts from various parts of the world, including known salts such as Himalayan, Maldon and Celtic. Therefore, although the amount of Li in Delice NSS is low, it is advantageous over refined table salt that does not contain Li and many other salts.

Strontium (Sr) is one of the rare elements found in soil, groundwater and human tissues and proven by scientific studies to be important for human bone health. The biological effects of Sr in the human body are related to its chemical similarity to calcium. Due to its similarity to calcium and its bone-seeking behavior, Sr has been reported to accumulate highly in bone and promote bone growth at certain concentrations (Usuda et al. 2007). It has also been reported that a daily intake of 680 mg of Sr reduces the risk of bone fractures by up to 33% (Canada 2019). The amount of Sr detected in Delice NSS is 37.11 ± 2.9 mg/kg. Although this amount is very small for daily intake, it is relatively more advantageous than refined table salt and other salts that do not contain Sr. In many studies on natural salts, Sr mineral is not mentioned, but because sea waters are rich in Sr, it is likely that sea salts also contain Sr (Fenner and Wright 2014).

350.2 ± 41.6 µg/kg Vanadium (V) was detected in Delice NSS. The amount of V in the soil depends on the type of parent rock. V is found in high amounts in tundra podzol and clay soil types. In addition, basic rocks contain higher amounts of V than neutral and acidic rocks. V is considered one of the essential trace elements in humans and animals. The daily need for V for the adult human body is 10–60 µg. For example, vanadyl sulfate, which is the sulfate salt of V, is a supplement used by bodybuilders to increase performance (Fawcett et al. 1997). It has also been reported that V may play a role in regulating the enzymes that control sodium in the human body (Mukherjee et al. 2004).

Zinc (Zn), which has been proven to be very important on human health in recent years, was detected in 44.0 ± 3.2 mg/kg of Delice NSS. In the TFC Supplementary Food Communiqué, the maximum daily intake limits are specified as 7.5 mg for the 4–10 age group, and 15 mg for the 11 year-old and over. According to EC and European Food Safety Authority (EFSA), daily Zn intake varies between 6.2 and 16.3 mg depending on body weight in adults, while these values vary between 2.9 and 14.2 mg for infants and children. Zn, which is responsible for the structure and catalytic activity of hundreds of enzymes in the human body, is mostly found in the muscles and bones (Cheng and Chen 2021). In its absence, clinical cases such as growth retardation, cell-mediated immune dysfunction and cognitive impairment have been reported. In addition, Zn deficiency has been observed in patients with chronic diseases such as malabsorption syndrome, liver disease, chronic kidney failure, sickle cell disease (Prasad 2013).

In addition to all these minerals in the structure of Delice NSS, Ca, Mg, and K minerals, which are indisputably very important for the human body, were determined and their concentrations were measured as 6160.16 ± 140.11 mg/kg, 48.26 ± 7.16 mg/kg, and 11.22 ± 5.6 mg/kg, respectively. Unlike refined table salts, natural rock and spring salts are rich in Ca, Mg and K minerals. In his study on 18 different natural salts, Ercoşkun (Ercoşkun 2022) reported that the Ca and Mg contents of the salts were 600–4000 mg/kg and 40–175 mg/kg, respectively.

The EC, Food and Drug Administration (FDA) and WHO have classified metals such as As, Cu, Cd, Hg, and Pb as heavy metals and/or toxic elements. In the technical specification guide for iodized salt published by WFP and the International Food Standards (IFS) Codex, the maximum permissible concentrations of these heavy metals for food grade salt are 0.5 mg/kg, 2.0 mg/kg, 0.5 mg/kg, 0.1 mg/kg and 1.0 mg/kg, respectively. In the TFC salt communiqué, the maximum value for Pb is stated as 2.0 mg/kg, which is different from WFP and IFS. Considering these levels, no heavy metal was detected in the content of Delice NSS at a level that would pose a risk (Table 1).

### Anion analysis by IC

**Fig. 1:**
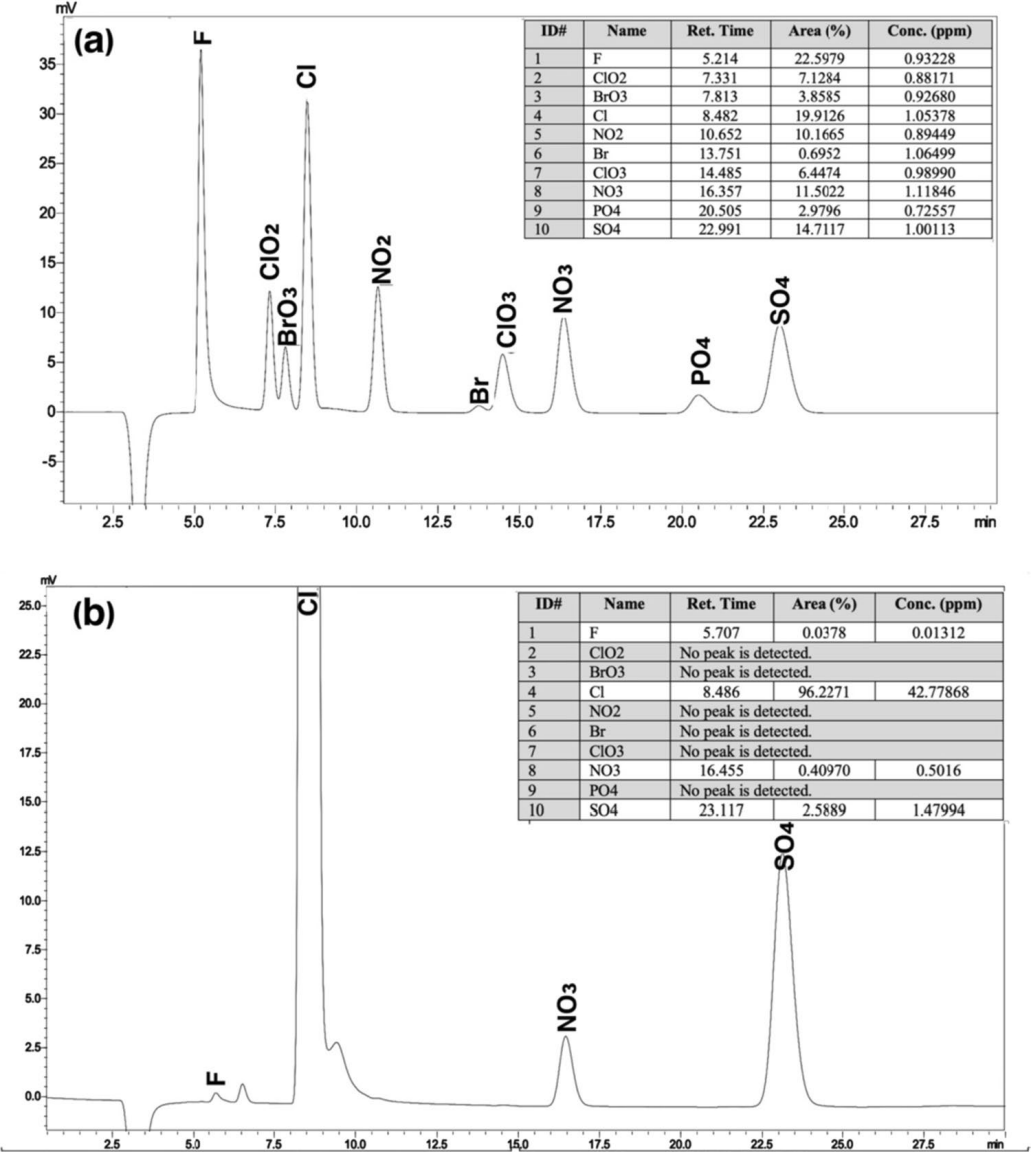
Anion chromatograms of standard solution (**a**) and Delice NSS (**b**)

Ion chromatography (IC) is a form of liquid chromatography in which atomic and molecular ions (anions and cations) are detected and separated using ion exchange resins. ASTM, ISO, US EPA and many other standards and regulatory bodies have approved IC-based ion analysis methods for food, drinking water and wastewater (Yelampalli et al. 2023). The presence of anions such as bromate, sulphate, chlorate, chlorite, phosphate, nitrate, nitrite, fluoride, and bromide in Delice NSS was investigated and the chromatograms of the results are shown in Fig. 1. Before analyzing the salt, a standard solution containing 1.0 mg/L concentration of each anion was analyzed and results were obtained with high accuracy (Fig. 1a). As a result of the analysis of Delice NSS, it was determined that it contained F^−^, SO_4_^−2^ and NO_3_^−^ ions and their concentrations were 0.013 mg/L, 0.409 mg/L and 1.479 mg/L, respectively (Fig. 1b). Among these anions, sulfate (SO_4_^−2^) is one of the components naturally present in food grade salts along with NaCl and included in the contaminant class. While sodium sulfate (Na_2_SO_4_) in its water-soluble form causes salt to absorb moisture, the presence of sulfate in the form of water-insoluble calcium sulfate can cause precipitation in foods. calcium sulfate (CaSO_4_) has also been reported to be an important risk factor for stomach cancer. Therefore, excessive amounts of sulfate in food-grade salts not only reduce the quality of the salt, but also cause various health problems (Heydarieh et al. 2020). In its technical specification for food-grade iodized salt, WFP refers only to the sulphate anion and states that it should be at a maximum level of 0.5% (w/w) (Guide 2020). According to WHO, the permissible limits of fluoride and nitrate in drinking water are 1 mg/L and 50 mg/L, respectively (Ward et al. 2018). Therefore, the levels detected in Delice NSS do not pose any risk to human health.

### CHNS elemental analysis

CHNS Elemental analysis (EA) is a sensitive technique used to simultaneously determine the total amount of carbon (TC), hydrogen (TH), nitrogen (TN), sulphur (TS) and oxygen (TO) in the structure of many materials. As a result of CHNS elemental analysis of Delice NSS, C and N were not detected, while H and S values were 0.191 ± 0.032% and 0.023 ± 0.002%, respectively. The detected H and S may be due to moisture and sulphate ion in the salt content. Moisture analysis and ion chromatography results support this possibility.

### ED-XRF analysis

**Fig. 2:**
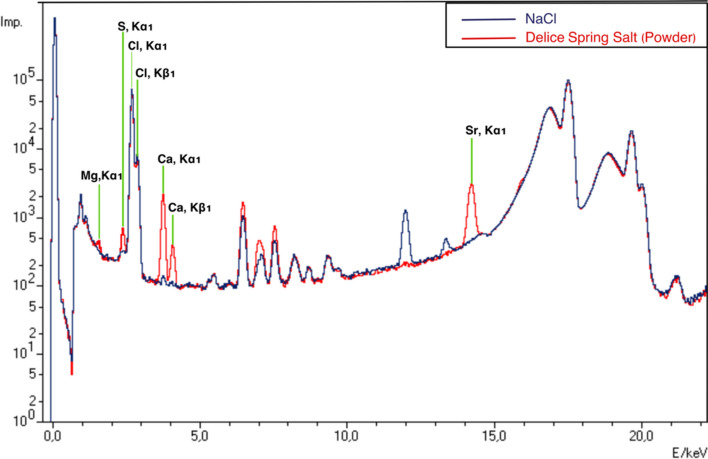
ED-XRF spectra of NaCl and Delice NSS

Energy dispersive X-ray fluorescence (ED-XRF) method is used for qualitative and quantitative analysis of many elements from sodium to uranium in a wide variety of applications such as food, petroleum, metal, polymer, plastic, cement, and mining. Some researchers have used XRF to determine the amounts of Na and some trace elements in foods (Pashkova 2009). In addition, Barakat et al. (Barakat et al. 2015) reported that the mineral contents of different commercial salts were determined with high accuracy by XRF. In this study, NaCl and Delice NSS were analyzed by ED-XRF under the same conditions and the spectrum is shown in Fig. 2. When the spectra are analyzed, the peaks at 2.622 keV and 2.815 keV for both salts are the characteristic K-alpha1 (Kα1) and K-beta1 (Kβ1) emission energies of Cl. Unlike NaCl, the energy peaks of 1.253 keV, 2.307 keV, 3.691 keV, 4.0112 keV and 14.163 keV in the spectrum of Delice NSS are the characteristic emission energies of Mg(Kα1), S(Kα1), Ca(Kα1), Ca(Kβ1) and Sr(Kα1), respectively (Barakat et al. 2015). Thus, the XRF results support the S determined by CHNS EA and the ICP-OES findings.

### Chemical bond analysis by FTIR

**Fig. 3:**
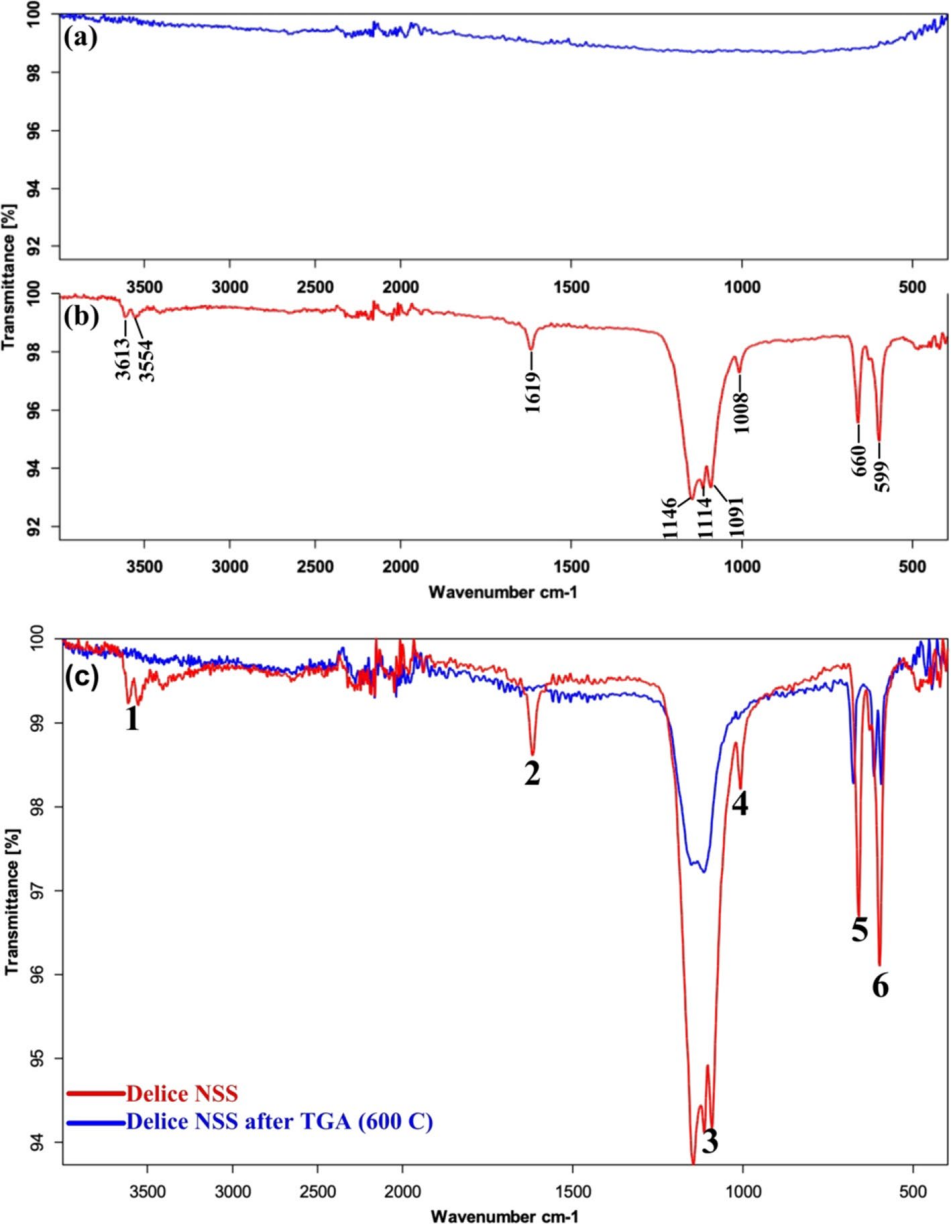
FTIR spectra of pure NaCl (**a**), Delice NSS (**b**), Delice NSS before and after heating to 600 °C (**c**)

The spectra of NaCl and Delice NSS characterized by FTIR are shown in Fig. 3. No absorption peak was observed in the spectrum of NaCl (Fig. 3a). In the literature, it is reported that salts such as LiCl, NaCl, KCl, CsCl, MgCl_2_, NaBr, KBr, NaI and KI do not absorb IR light in the mid infrared region (MIR, 400–4000 cm^−1^) (Max et al. 2001). In contrast, inorganic salts containing many polyatomic ions such as nitrate, carbonate, phosphate, chlorate, bromate, thiocyanate and sulphate exhibit characteristic IR absorption bands. Miller and Wilkins (Miller and Wilkins 1952) reported the IR spectra and characteristic absorption frequencies of 159 pure salts containing polyatomic ions. Considering their research and the polyatomic sulphate and nitrate ions detected by IC in this study, the 3613 cm^−1^, 3554 cm^−1^, 1619 cm^−1^, 1146 cm^−1^, 1114 cm^−1^, 1091 cm^−1^, 1008 cm^−1^, 660 cm^−1^ and 599 cm^−1^ vibrational peaks observed in the FTIR spectrum of Delice NSS (Fig. 3b) may be attributed to sulphate and nitrate compounds of Ca, Mg and Sr. Thus, FTIR characterization revealed that Delice NSS is not only composed of NaCl.

### Thermal analysis

**Fig. 4:**
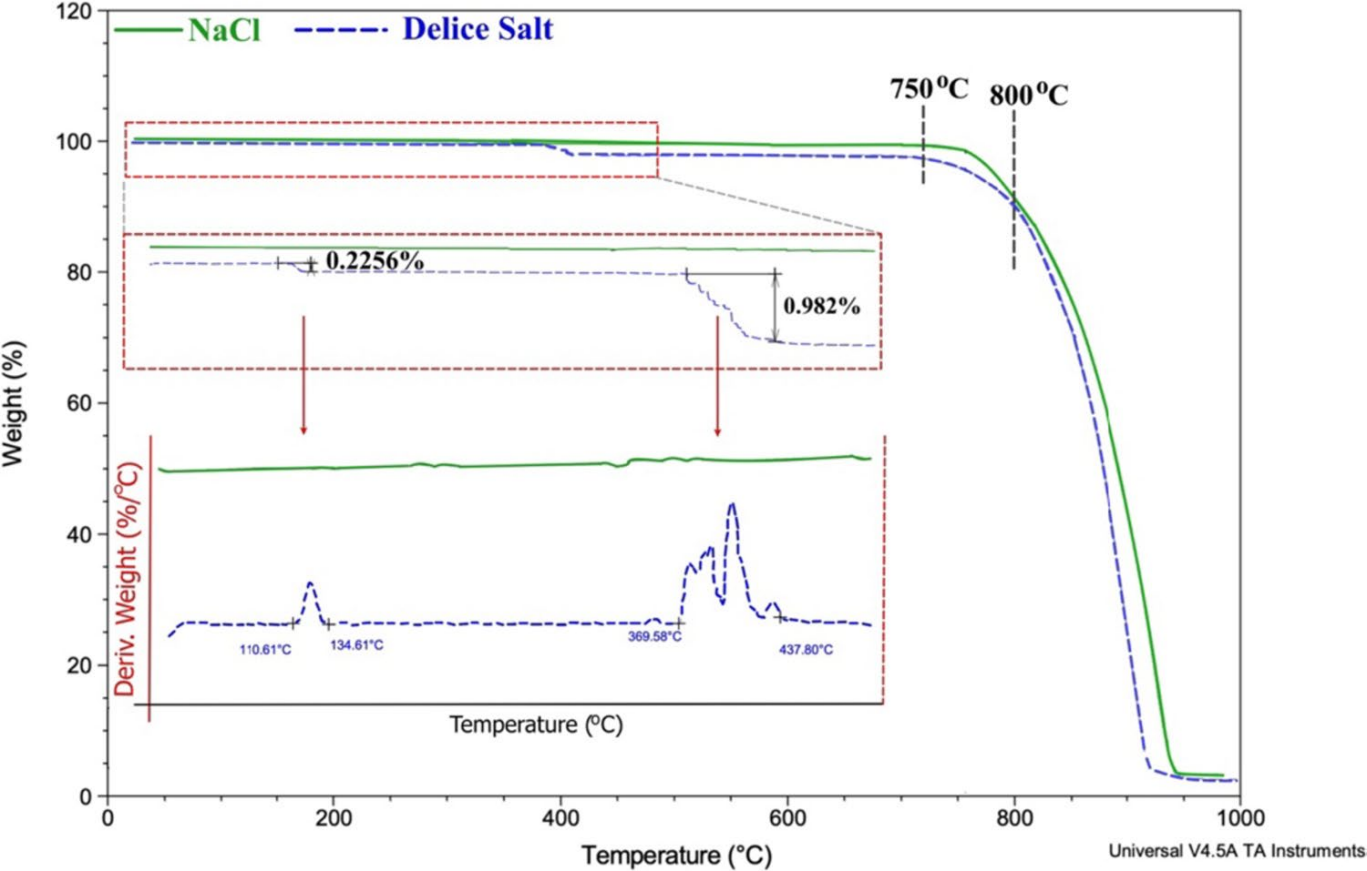
TGA/DTG thermograms of NaCl and Delice NSS

TGA thermograms showing the mass changes of NaCl and Delice NSS as a function of temperature are given in Fig. 4. While no mass change was observed in NaCl in the 30–700 °C temperature range, a two-step change was observed in Delice NSS. The mass loss of 0.2256% between 110 and 135 °C is due to the moisture in the structure of the salt. The mass loss of 0.982% between 350 and 450 °C may be due to the decomposition of molecular ions such as sulphate and nitrate in the salt structure (Xu et al. 2019). Mass loss occurs due to NO_x_, SO_x_ and O_2_ gases released during decomposition. For both salts, a slow mass loss due to sublimation occurred between 750 and 800 °C, while intense evaporation started above 800 ºC, which is close to the melting point (801 °C) of NaCl (Wang et al. 2018).

### Investigation of microplastic content

**Fig. 5:**
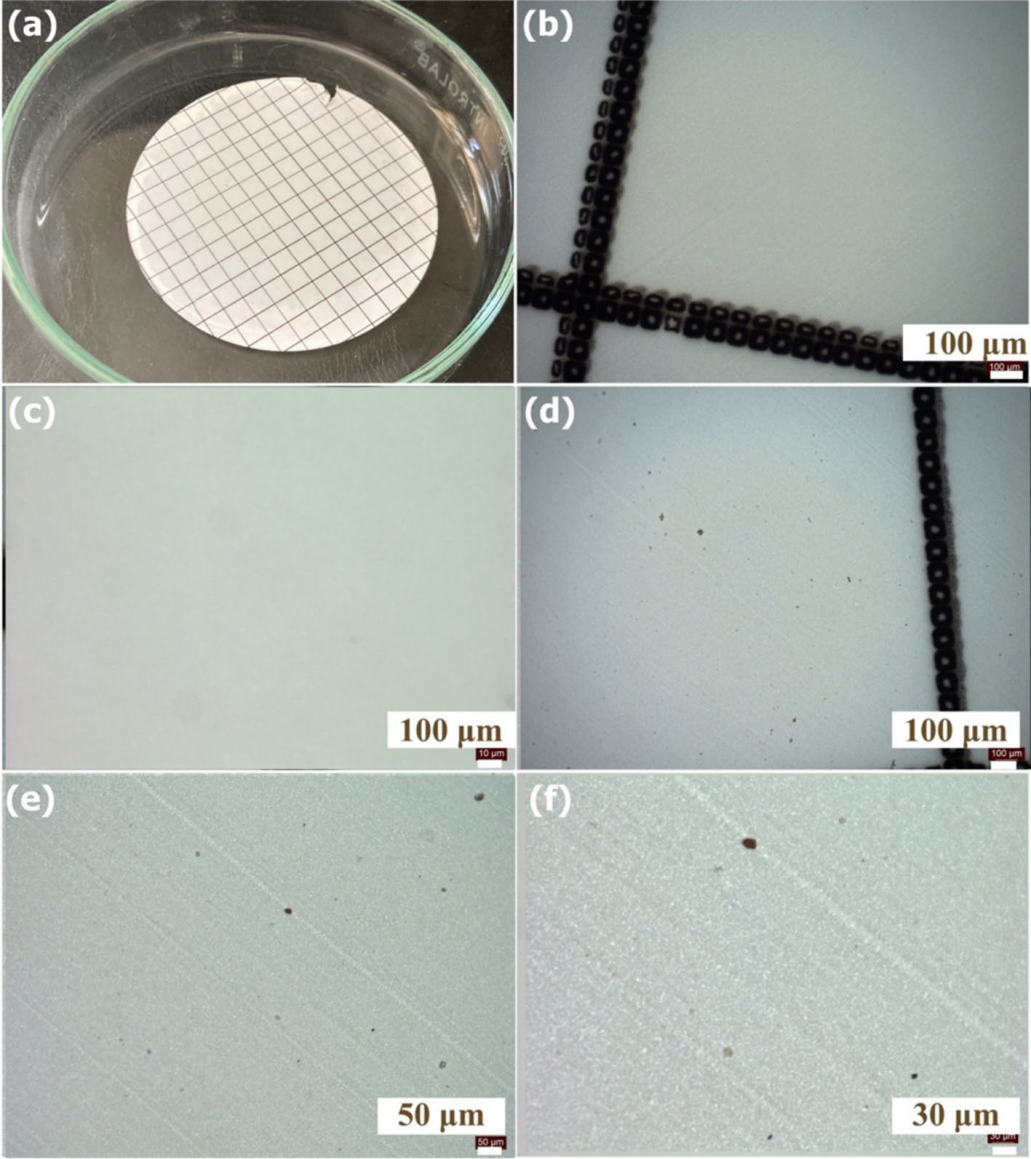
Images of Delice NSS under microscope after extraction: Scale filter paper (pore size 0.45 m) (**a**), filter images with ultrapure water only (**b, c**), filter paper images with Delice NSS solution (**d–f**)

Plastic wastes with a particle size between 0.1 μm and 5 mm have been classified as MPs (Ali et al. 2023). MPs have been assumed to be toxic pollutants due to their chemical stability and non-biodegradable properties. Recently, they have become an increasing research topic in air, water, soil, and food (Ali et al. 2023, Ercoşkun 2022). The microscope images of the filter papers through which dH2O and Delice NSS extract were passed are shown in Fig. 5. While no noticeable particles were detected only on the filter paper through which dH2O was passed, some particles smaller than 30 μm in size were detected in the image of Delice NSS. However, further characterization methods are needed to determine whether these particles are MPs. For this reason, spectroscopy and thermal analysis methods have recently been used to detect MPs (Costa-Gómez et al. 2023). FTIR spectrometry is one of the most common methods used for the detection and identification of microplastics. In the FTIR spectra of plastics produced from polymers such as PE, PET, PVC, PP, PS, and nylon, which are widely used in daily life, sharp absorption peaks appear in the region between 2800 and 3000 cm^−1^ due to C–H stretching vibrations (Bozkaya 2023). However, no peak in this region appeared in the FTIR spectrum of Delice NSS, which may support that it does not contain MPs. In addition, it has been reported in the literature that all of the above-mentioned polymers except PET undergo complete degradation at the end of 600 °C (Devasahayam et al. 2019). Therefore, in order to determine whether the degradation occurring between 350 and 450 °C in the Delice NSS TGA thermogram belongs to a plastic, Delice NSS was heated to 600 °C and then analyzed again by FTIR (Fig. 3c). When Fig. 3c is examined, it is seen that the peaks numbered 1,2 and 4 disappeared and the peak groups numbered 3,5 and 6 showed differences and decreased intensities. Since not all peaks disappear completely, this phenomenon may be attributed to the decomposition of nitrate and/or sulphate compounds. Because Goedecke et al. used simultaneous FTIR-TGA method in addition to other methods to detect microplastics belonging to polymers such as PE, PP and PS and reported that all peaks disappeared in the FTIR spectrum after 600 °C (Goedecke et al. 2020).

## Conclusion

Natural rock and spring salts have become the favorite of world-famous kitchens and gourmets because they do not contain chemical additives, are rich in minerals and have their own unique flavors. In this study, physicochemical characterization of the solid salt form obtained by evaporation of salty water coming to the surface in the form of spring water in Delice region in Kırıkkale province of Turkey was carried out. The NaCl, suspended solid moisture and acid insoluble matter contents of Delice NSS were analyzed and determined as 98.79% (w/w), 0.301% (w/w), 0.16% (w/w) and 0.01% (w/w), respectively. As a result of mineral analyses performed by ICP-OES, elements such as Mg, K, Ca as well as rare elements Li, Sr and V were determined. IC analyses also revealed that Delice NSS contains fluoride, nitrate, and sulphate anions. All results were compared with the criteria of the European Commission, World Health Organization, World Food Program, International Food Standards, Turkish Food Codex and Turkish Salt Codex and it was concluded that the results did not exceed the limits specified in these standards. The chemical and thermal properties of Delice NSS were characterized by Fourier transform infrared spectroscopy (FTIR), CHNS elemental analyzer, X-ray fluorescence (XRF) and thermogravimetric analyzer (TGA) and the results were compared with pure NaCl. The FTIR characterization revealed that Delice NSS is not only composed of NaCl. As a result of CHNS elemental analysis of Delice NSS, C and N were not detected, while H and S values were 0.191 ± 0.032% and 0.023 ± 0.002%, respectively. In the TGA analysis, while no mass change was observed in NaCl in the temperature range of 30–700 °C, a two-stage mass loss was observed in Delice NSS. XRF analysis confirmed that Delice NSS contains Mg, Ca and Sr elements and differs from pure NaCl. Even though some particles were detected on the filter paper through which the Delice NSS extract was passed, it was concluded that these particles were not microplastic by TGA and FTIR analyses. The results reveal that Delice NSS contains a variety of minerals compared to pure NaCl and is also free of microplastics. Although it does not contain contaminants such as heavy metals and microplastics, a hazard or risk analysis is recommended to determine whether it poses a risk as a food ingredient.

### Supplementary Information

Below is the link to the electronic supplementary material.

Supplementary file1 (DOCX 460 KB)

## Data Availability

The data that support the findings of this study are available from the corresponding author, [Ogün Bozkaya], upon reasonable request.
