# Supplementary material for: Physico-chemical characterization of food grade natural spring salt from the Central Anatolia region of Turkey and investigation of its microplastic content
Source: J Food Sci Technol. 2024 Feb 26;61(9):1711–21. doi: 10.1007/s13197-024-05942-0 (PMC11263517; doi:10.1007/s13197-024-05942-0)
Supplement: Supplementary file 1 — Supplementary file1 (DOCX 460 KB) [file 13197_2024_5942_MOESM1_ESM.docx]

**
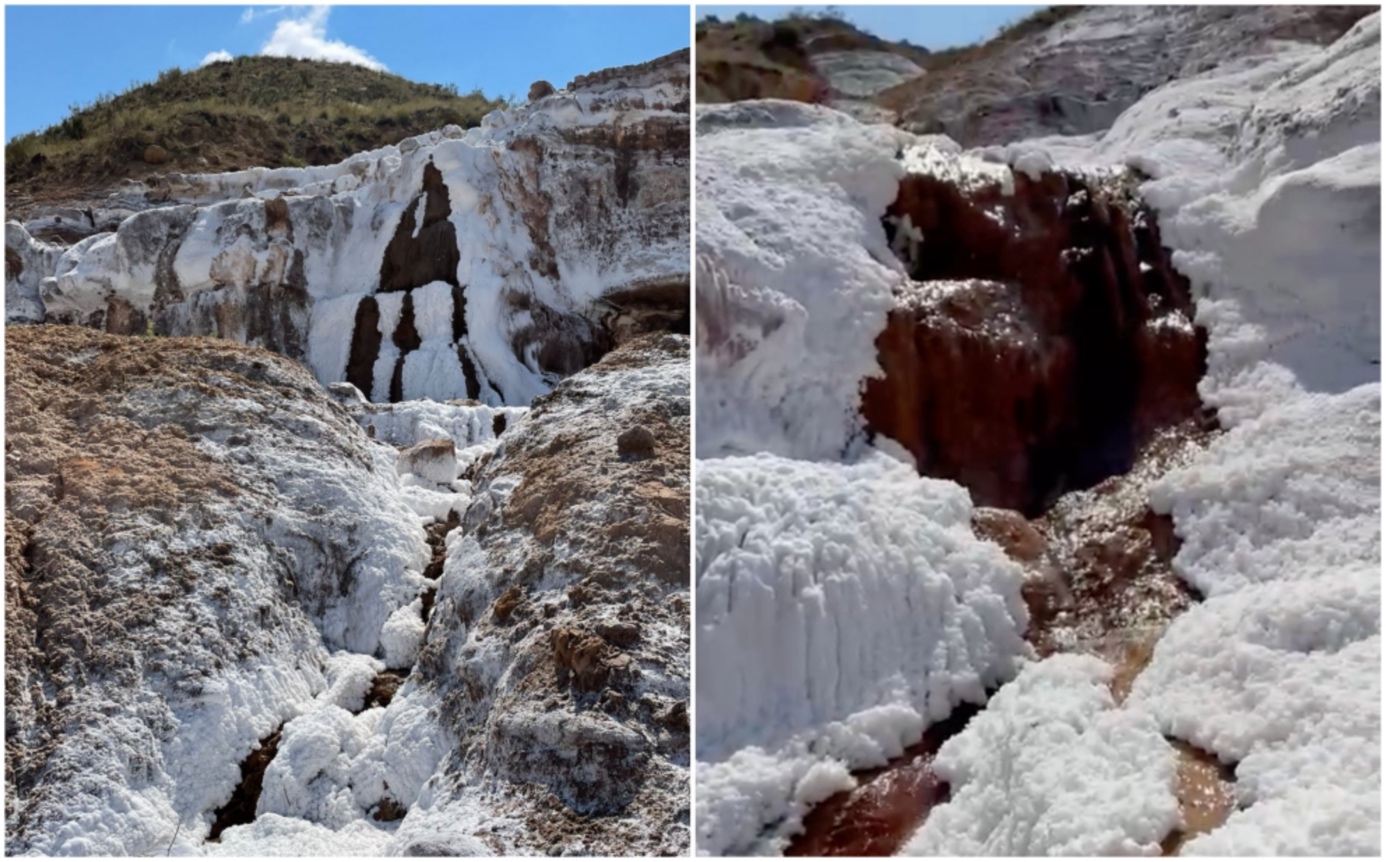
**

**Fig. 1** Delice natural spring salt.

**Physicochemical characterization**

**Determination of %NaCl content**

The percentage of NaCl of Delice NSS was calculated according to ASTM D632-18. In this context, 10.00±0.01 g of the salt sample, which was pulverized in a mortar, was taken into a beaker and 250 mL of distilled water was added to it. Then, 10 mL of 1/4 diluted nitric acid solution was added to the solution, and the solution was stirred at room temperature for 20 minutes. After all the solid had dissolved, the solution was transferred to a 2 L flask and distilled water was added up to the volume line. With a pipette, 25 mL of solution was taken and transferred to a beaker. 0.5 g of calcium carbonate (CaCO_3_) was added to the solution to neutralize excess HNO_3_, and its pH was adjusted to about 7. Immediately after, 3 mL of potassium chromate (K_2_CrO_4_) solution was added to the adjusted solution as an indicator and titrated with AgNO_3_ dropwise until the solution color became permanent yellowish-brown. After titration, the total chloride, expressed as a percentage of NaCl, was calculated by the following equation:

$$P=\left[ \left( A/B \right) x \left( C/D \right) \right] x 100$$

where A is reagent grade NaCl used (g), B is 0.05 N AgNO_3_ solution required to titrate the reagent grade NaCl (mL), C is 0.05 N AgNO_3_ solution required to titrate the sample being tested (mL), D is test sampling mass (g) and P is total chlorides, expressed as sodium chloride, in the sample being tested (%).

**Determination of total solids**

The glass petri dish was brought to constant weight at 105 ºC. Then, 5.0 g of salt sample was put into it and kept at 105 ºC for 2 hours. After the sample taken from the oven was cooled for a while in the desiccator, it was weighed with a precision balance. In order to ensure that it came to constant weight, the sample was put in the oven again and left for 30 minutes. After the waiting period, the amount of evaporation was calculated by weighing again.

**Determination of suspended solid matter**

After it was kept in an oven at 105 ºC for 1 hour and cooled in the desiccator, 0.45 μm filter paper with constant weight was placed in the filtration mechanism. It was wetted with distilled water and passed under vacuum, then a well-mixed 5.0 g salt/100 ml water solution was filtered. After the filtration process was completed, the walls of the funnel were thoroughly washed with distilled water and the vacuum was continued for a while. The filter paper was carefully taken with the help of forceps, placed in a clean glass petri dish and dried in an oven at 105 ºC for 2 hours. Then, the amount of suspended solids was calculated by cooling in a desiccator and weighing.

**Determination of acid insoluble matter**

First, a glass gooch crucible was washed with hydrochloric acid and then with hot water until the chloride did not react. Then it was dried in an oven at 105 ºC and weighed. Then, approximately 20 g of the salt sample was dissolved in a 200 mL beaker containing 10% HCl. After boiling the solution for 5 minutes, it was left to stand for 30 minutes. The resulting solution was then filtered through a glass gooch crucible. After the filtration process was completed, the gooch crucible was washed 5 times with boiling 10% HCl and hot distilled water, respectively. It was checked whether the filtrate gave chloride reaction with AgNO_3_. After washing, the gooch crucible was dried in an oven at 105 ºC until it reached a constant weight. Then, it was placed in a desiccator and allowed to cool, and then weighed again to calculate the amount of acid insoluble matter.

**Result and Discussion**

**Sodium and other trace element analysis by ICP-OES**

Drake and Drake reported the Na content of many sea and rock salts around the world (Table 1). When Table 1 is examined, it is seen that Delice NSS contains less sodium than many salts (Drake & Drake, 2011).

**Table 1.** The amount of Na in the content of some salts on Earth

| **Salt** | **Na (ppmx10^5^)** | **Location** | **Salt** | **Na (ppmx10^5^)** | **Location** |
| --- | --- | --- | --- | --- | --- |
| Kala Namak | 3.66 | India | Light Grey Sea | 3.66 | France |
| Bolivian Rose | 3.70 | Andes Mount. | Atlantic Sea | 3.38 | Portugal |
| Himalayan Pink | 3.68 | Himalayan Mount. | Redmond Realsalt | 3.69 | Utah, USA |
| Paphaku White | 3.21 | Hawaii | Frontier Sea | 4.37 | Northern French Atlantic |
| Haleaka Red | 3.09 | Hawaii | Bali Reef Sea | 4.69 | Bali |
| Kilauea Black | 3.03 | Hawaii | Sal Grosso | 4.37 | Brazil |
| Palm Island Red G. | 3.59 | Hawaii | Cyprus Silver Sea | 4.08 | Cyprus |
| White Silver | 3.63 | Hawaii | Flor de Sal de Manzanilla | 3.98 | Mexico |
| Palm I. Bamboo J. | 3.66 | Hawaii | Korea Bay Gray | 4.49 | Korea |
| Black Lava | 3.66 | Hawaii | Moroccan Atlantic | 4.43 | Morocco |
| Pacific Natural Sea | 3.83 | New Zealand | Okinawa Shima Masu Sea | 3.73 | Japan |
| New Z. Organic Sea | 3.76 | New Zealand | Vietnamese Pearl Sea | 4.43 | Vietnam |
| Fleur de Sel de C. | 3.59 | France | Shinkai Deep Sea | 4.39 | Japan |
| Le Balaine | 4.02 | France | Sal Marine de Noirmouteir | 4.41 | Portugal |
| Fluer de Sel de G. | 3.79 | France | Ittica d’Or Sicilian Sea | 4.02 | Italy |
| Halon Mon | 3.65 | Wales, U.K. | Sonoma | 3.91 | West Coast, USA |
| Maldon | 3.83 | England | Murray River | 3.66 | Australia |

**References**

Drake, S., & Drake, M. (2011). Comparison of salty taste and time intensity of sea and land salts from around the world. *Journal of Sensory Studies*, *26*(1), 25-34.
